# Dual targeting carbonic anhydrase inhibitors as promising therapeutic approach: a structural overview

**DOI:** 10.3389/fmolb.2025.1511281

**Published:** 2025-02-03

**Authors:** Katia D’Ambrosio, Anna Di Fiore, Emma Langella

**Affiliations:** Institute of Biostructures and Bioimaging - CNR, Napoli, Italy

**Keywords:** dual targeting compounds, carbonic anhydrase inhibitors, crystallography, multifactorial diseases, drug design

## Abstract

The dual-target inhibitor strategy is an evolving approach that holds great potential for treating complex diseases by addressing their multifactorial nature. It can enhance therapeutic outcomes, reduce side effects and avoid the emergence of drug resistance, particularly in conditions like cancer, inflammation and neurological disorders, where multiple pathways contribute to disease progression. Identifying suitable targets for a dual inhibitor approach requires a deep understanding of disease biology, knowledge of critical pathways, and selection of complementary or synergistic targets. Human carbonic anhydrases (hCAs) have been recognized as suitable drug targets for this therapeutic approach. These enzymes play a key role in maintaining pH balance, ion transport, and fluid regulation across various tissues and organs and their dysregulation has been associated to a variety of human pathologies. Consequently, the inhibition of hCAs combined to the possibility to modulate the activity of a second molecular target represents a promising way for developing more effective drugs. In this mini-review, we aim to present an overview of the most significant structural results related to the development of novel therapeutics employing hCA inhibitors as dual-targeting compounds for the treatment of complex diseases.

## 1 Introduction

Human carbonic anhydrases (hCAs) are zinc-containing metalloenzymes that catalyze the reversible hydration reaction of carbon dioxide to bicarbonate and proton (Supuran, 2023). To date, fifteen distinct hCA isoforms have been identified, which vary in their oligomeric structures, distribution across different organs and tissues, subcellular localization, and catalytic performance (Alterio et al., 2012; Di Fiore et al., 2020; Mishra et al., 2020). These enzymes play a crucial role in numerous physiological functions, and disruptions in their expression or activity have been linked to a variety of human disorders, such as epilepsy (hCA II, VII, XIV), obesity (hCA VA, VB), and cancer (hCA II, IX, XII) (Langella et al., 2018; Langella et al., 2021; Supuran et al., 2018; Supuran, 2021). Consequently, hCAs have become significant targets for drug development (Angeli et al., 2020), with ongoing research focused on discovering selective inhibitors for specific isoforms implicated in different diseases.

Dual targeting inhibitors represent a promise as a viable therapeutic strategy in drug design, aiming to modulate simultaneously two different targets or pathways within a disease context. This approach is particularly advantageous when redundant or compensatory pathways limit the effectiveness of single-target therapies, since it can lead to an increased drug efficacy by preventing drug resistance development, reducing the required dosage of single drugs, and limiting the risk of side effects (Lopez and Banerji, 2017; Makhoba et al., 2020; Wang and Tortorella, 2022).

This strategy has been widely employed also in the case of hCAs, leading to the design of compounds able to inhibit hCA alongside another target. In particular, in the last years many studies focusing on dual targeting inhibitors of hCA enzymes were reported for the treatment of complex diseases, including cancer, inflammatory conditions, glaucoma and neurological disorders (Meleddu et al., 2018; Mincione et al., 2021; Angeli et al., 2023b; Ronca and Supuran, 2024). Numerous studies rely on *in vitro*, cellular, and *in vivo* assays to demonstrate the effectiveness of these compounds. However, from a structural standpoint, there is limited information in the literature on how these compounds interact with their specific targets, and the few available data are scattered. Nevertheless, such insights are essential for designing new compounds with enhanced properties.

In this Mini Review, we aim to report the existing structural information obtained through crystallographic studies to provide an updated structural overview of state-of-the-art results in this research field. In particular, we will present the various structural data available for the most physiologically relevant hCA II isoform in complex with compounds acting as dual-targeting agents and group them on the basis of related pathologies.

## 2 Dual inhibitors used as anti-glaucoma agents

**FIGURE 1:**
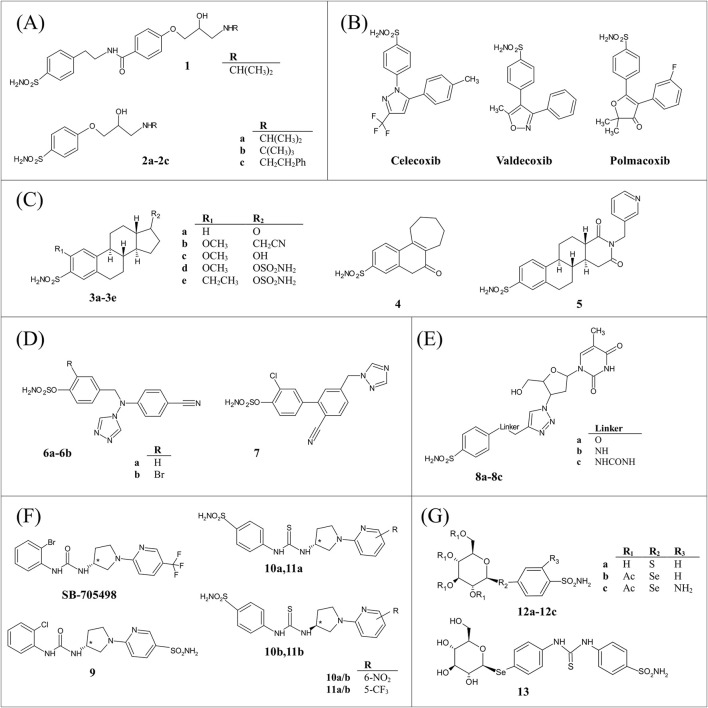
Chemical structures of dual inhibitors used as anti-glaucoma agents **(A)**, anti-inflammatory agents **(B)**, anti-cancer agents **(C–E)**, and dual targeting compounds for treating neurological diseases **(F, G)**.

Among the dual targeting hCA inhibitors (hCAIs), some interesting molecules are those designed in 2018 by Nocentini et al which are utilized for the treatment of glaucoma (Nocentini et al., 2018). A β-adrenergic receptor (AR) blocker combined with a hCAI in eye drops is one of the clinical options available for antiglaucoma therapy. Indeed, both molecules decrease intraocular pressure (IOP) reducing the production of aqueous humor, the first by blocking the sympathetic nerve endings in the ciliary epithelium (Brooks and Gillies, 1992), and the second by slowing the rate of bicarbonate production and the consequent reduction of the transport of water and osmotically obligated sodium within the fluid (Masini et al., 2013). The approach proposed by Nocentini consists in designing compounds which possess two functional groups able to interact concomitantly with the two target enzymes. In particular, the benzenesulfonamide moiety represents the hCA inhibitory fragment, while the aryloxy-2-hydroxypropylamine portion that of β-blockers. Two subgroups of molecules were designed, one in which the aryloxy-2-hydroxypropylamine portion was detached with an ethylbenzamide spacer to the benzenesulfonamide scaffold (compound **1**), and another where the two pharmacophores were directly attached to each other (compounds **2a-2c**) (Figure 1A).

**FIGURE 2:**
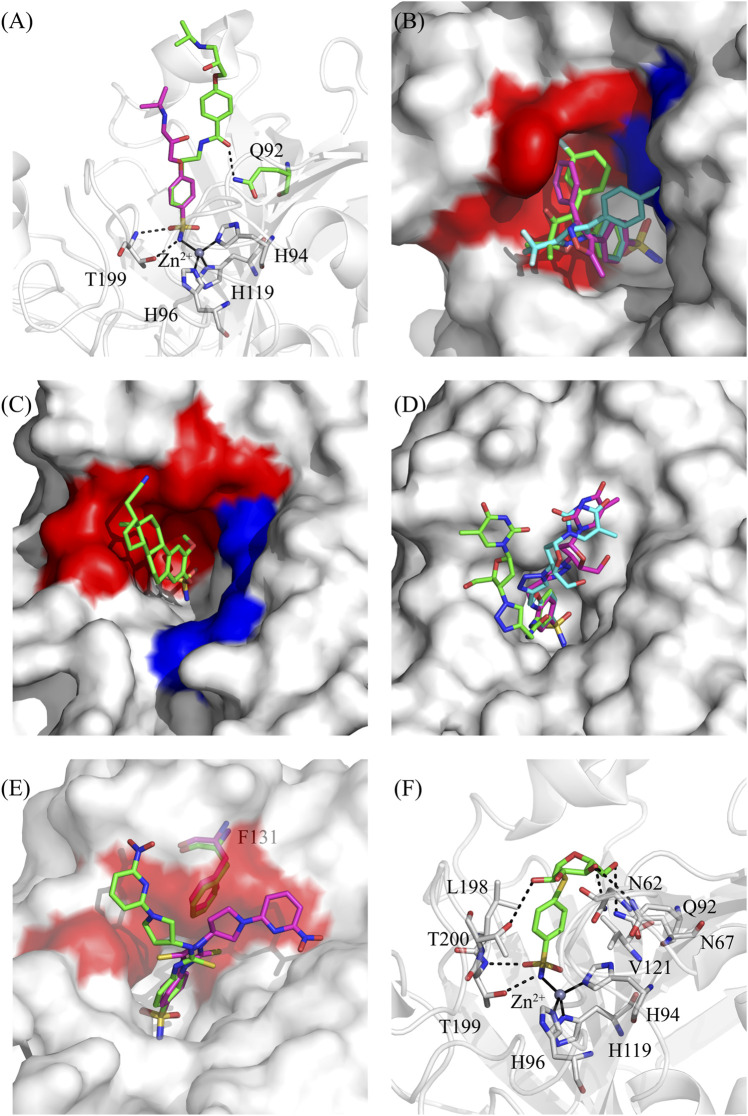
X-Ray structures of representative dual inhibitors bound to hCA II. **(A)** Superposition between hCA II/**1** (green, PDB code 5WLV) and hCA II/**2a** (magenta, PDB code 5WLT). The hydrogen bond between compound **1** and Gln92 is shown as a dotted black line. Zinc ion coordination and hydrogen bond interactions between Thr199 and sulfonamide moiety are also reported. **(B)** Superposition of **celecoxib** (cyan, 1OQ5 PDB code), **valdecoxib** (magenta, 2AW1 PDB code) and **palmacoxib** (green, 5GMN PDB code) in complex with hCA II. The protein accessible surface is shown with hydrophobic residues (Ile91, Val121, Phe131, Val135, Leu141, Leu198) coloured in red, and hydrophilic ones (Asn67, Glu69, Gln92) in blue. **(C)** Accessible surface of hCA II in complex with compound **3b** (PDB code 3BET), chosen as representative inhibitor of hCA/STS-dual inhibitors. Residues delimiting the hydrophobic and hydrophilic regions of hCA II active site cavity were coloured in red and blue, respectively. **(D)** Superposition of hCA II bound to dual inhibitors targeting hTL: hCA II/**8a** (cyan, PDB code 6YPW), hCA II/**8b** (magenta, PDB code 6WKA) and hCA II/**8c** (green, PDB code 7NH6). Accessible surface of hCA II is also depicted. **(E)** Superposition between hCA II/(*R*)**-10a** (green, PDB code 8BJX) and hCA II/(*S*)**-10b** (magenta, PDB code 8BOE). Phe131 of both adducts and the double conformation of thioureido moiety observed for compound (*S*)**-10b** are shown. Accessible surface of hCA II is also depicted with residues delimiting the hydrophobic region of hCA II active site cavity in red. **(F)** Active site view of hCA II/**12a** (PDB code 7ZWB). Residues involved into ligand binding are depicted, with hydrogen bonds shown as dotted black lines. Zinc ion coordination is also reported. Figures were made by using PyMol. SASA was calculated with a default probe radius of 1.4 Å.

The resulting two series of compounds were investigated for their inhibitory activity against hCAs I, II, IX, and XII and for their effectiveness to modulate the β1- and β2-ARs. The first one exhibited a notable inhibitory potency against hCAs (K_Is_ 1.2–83.1 nM) at the expense of zero affinity to β-ARs, while the second one showed a slight worsening of hCA inhibition (K_Is_ 3.5–1174.3 nM), with the affinity for β-ARs increasing up to the micromolar range. The X-ray structures of compounds **1** and **2a** in complex with hCA II were also determined, showing that both sulfonamide moieties participate in the typical interactions of this class of hCAIs (Alterio et al., 2012). In particular, the ionized nitrogen atom is tetrahedrally coordinated to the zinc ion and is hydrogen bonded to Thr199-OG atom. An additional interaction between one oxygen of sulfonamide and Thr199-NH atom stabilizes the inhibitor binding (Figure 2A). Both compounds form several van der Waals interactions, but only compound **1** establishes also a hydrogen bond with Gln92 side chain, thus explaining its higher binding affinity for hCA II compared to compound **2a** (Nocentini et al., 2018).

Some representative compounds selected among the best dual-inhibitors of this hCAI class, i.e., **2a-2c**, were also evaluated for their IOP lowering properties in a rabbit model of glaucoma. Notably, they induce the higher reduction of IOP with respect to the clinically used dorzolamide, timolol, and their combination, demonstrating that β-AR blocker–hCAI hybrids are potential candidate drugs for antiglaucoma therapy with a novel mechanism of action (Nocentini et al., 2018).

## 3 Dual inhibitors as anti-inflammatory agents

In the last years many efforts have been dedicated to develop Cyclooxygenase-2 (COX-2) specific inhibitors with the aim to improve the therapeutic potency and reduce the gastro-intestinal side effects of classical non-steroidal anti-inflammatory drugs (NSAIDs), which instead inhibit both COX-1 and COX-2 isozymes (Scott and Lamb, 1999; de Leval et al., 2000; Croom and Siddiqui, 2009; McCormack, 2011).

Some of these molecules incorporate in their chemical structure a primary sulfonamide moiety, which is the key mediator of the interaction of these agents also with several hCA isoforms (de Leval et al., 2002; Dogné et al., 2005; Scozzafava et al., 2004; Supuran et al., 2004; Supuran and Scozzafava, 2002). This ability has been considered an important factor for the reduction of side effects of these NSAID agents, since it has been hypothesized that hCAs could act as “sponge”, decreasing COX-2 binding in the gastro-intestinal tract and kidney (Kim et al., 2016).

Three molecules of this type were biochemically and structurally characterized, i.e., **celecoxib**, **valdecoxib** and **polmacoxib** (Figure 1B) (Simon et al., 1999; Supuran et al., 2004; Weber et al., 2004; Di Fiore et al., 2006; Kim et al., 2016).

**Celecoxib** and **valdecoxib** presented good inhibitory activity against both the physiologically most relevant isoform hCA II (K_I_ of 21 and 43 nM, respectively), and the two tumor-associated isoenzymes hCA IX (K_Is_ 16 and 27 nM) and hCA XII (K_Is_ 18 and 13 nM). In contrast, moderate potency was observed for the membrane-bound hCA IV isoenzyme and weak efficacy for hCA I (Di Fiore et al., 2006). In the case of **polmacoxib**, the obtained IC_50_ values were compared with those of **celecoxib**, showing that against hCA I **polmacoxib** presents a stronger inhibitory activity, while against hCA II its inhibitory activity was slightly less potent (Kim et al., 2016).

The crystal structures of these compounds in complex with hCA II were determined (Weber et al., 2004; Di Fiore et al., 2006; Kim et al., 2016), showing that even though in all the adducts the classical sulfonamide binding mode was observed (Alterio et al., 2012), the position of the tail moieties was different for each inhibitor (Figure 2B). In particular, the *m*-fluorophenyl of **polmacoxib** and the phenyl ring of **valdecoxib** pointed towards the same hydrophobic region of the protein active site, while were rotated by approximately 45° and 90°, respectively, with respect to the *p*-tolyl moiety of **celecoxib**, which was instead surrounded by hydrophilic residues, i.e., Asn67, Glu69, and Gln92. Moreover, the phenyl ring of **valdecoxib** formed a strong π-π interaction with Phe131, which instead was lost by *p*-tolyl group of **celecoxib** due to the 90° rotation, caused by steric hindrance between its methyl substituent and Phe131 and Ile92 residues. In **polmacoxib** the *m*-fluorophenyl moiety was located between the *p*-tolyl of **celecoxib** and the phenyl of **valdecoxib**, due to the fluorine atom in meta position. The peculiar characteristic of **celecoxib** of completely filling hCA II active site, with its trifluoromethyl group in the hydrophobic part of the cavity and the *p*-tolyl portion in the hydrophilic one, may explain why it is the most active hCA II inhibitor among this subclass (Di Fiore et al., 2006).

## 4 Dual inhibitors acting as anti-cancer agents

The therapeutic potential of molecules targeting tumor-associated hCA IX and XII has emerged in the last decade, with numerous studies reported on inhibitors able to affect several tumor features, such as intra-cellular pH, drug availability, and redox homeostasis cell cycle (Kumar et al., 2022; Ronca and Supuran, 2024). Due to the better cancer therapy outcomes predicted for a dual-targeting approach, investigations on hCAIs able to interact also with another cancer-related target were reported (Berrino et al., 2020; Elzahhar et al., 2020; Zhang et al., 2021; Hefny et al., 2024). At the moment, structural data on this type of dual-target inhibitors are available only for two classes of hCAIs, namely, aryl sulfamate-based molecules (Figure 1C) (Lloyd et al., 2005a; Lloyd et al., 2005b; Leese et al., 2006a; 2008; Woo et al., 2008; 2010a; Cozier et al., 2010a) and benzenesulfonamide derivatives containing an azidothymidine moiety (Figures 1D, E) (Berrino et al., 2020; Plyasova et al., 2021).

Compounds of type **3a-3e** were early used for the treatment of hormone-dependent breast cancer since they lead to a reduction of estrogenic steroids, responsible for the growth and development of this type of cancer, via the steroid sulfatase (STS) pathway (Reed et al., 2005; Stanway et al., 2006; Woo et al., 2008). Their exceptional properties *in vivo* seem to be related to their almost complete uptake by red blood cells after oral administration and consequent protection against first pass metabolism (Ireson et al., 2004). In particular, it has been suggested that hCA II binds to sulfamate moiety, influencing oral bioavailability and pharmacokinetics, thereby enhancing the overall efficacy of these compounds.

The 3-O-EMATE (compound **3a**) was the first and one of the most potent STS inhibitors to be characterized. However, *in vivo* studies showed that it was extremely estrogenic in rodents and consequently not suitable for clinical use (Elger et al., 1995). Starting from these results, other molecules were then designed, including tricyclic nonsteroidal analogues (compound **4**) (Lloyd et al., 2005a; Stanway et al., 2006) and EMATE derivatives obtained by modification of its scaffold and/or its substituent pattern (compounds **3b-3e** and **5**) (Lloyd et al., 2005b; Leese et al., 2006b; 2008; Woo et al., 2008; Cozier et al., 2010b). Inhibition assays revealed that this hCAI series includes compounds showing medium to high efficacy against hCA II (IC_50_ values in the range 0.1–770 nM), whereas poorer inhibition was observed in the presence of a substituent in position 2 (compounds **3b-3e)** (IC_50_ > 1500 nM).

Crystal structures of hCA II in complex with these sulfamate-based inhibitors indicated that this zinc binding group preserves all the interactions described above for its sulfonamide bioisoster. Interestingly, their steroidal backbone was accommodated into the hydrophobic portion of active site cleft establishing a large number of strong van der Waals interactions, thus stabilizing the inhibitor binding (Figure 2C).

Another suitable strategy to counteract hormone dependent tumors involves the reduction of estrogen levels by aromatase inhibition using molecules that contain as active pharmacophore a heme-chelating azole ring, such as triazole (Lloyd et al., 2005a; Woo et al., 2010b). The presence of both triazole moiety and sulfamate functionality on chemical scaffold (compounds **6a-6b** and **7**) leads to the development of very effective molecules possessing a multi-targeting mechanism of action (Figure 1D).

A different series of dual-inhibitors acting against tumour-associated hCAs and another challenging cancer target, namely, human telomerase (hTL), was recently characterized. hTL supports the unlimited proliferation of cancer cells and its catalytic subunit is highly expressed in the majority of hypoxic tumours (Harley, 2008; Wang et al., 2018). However, the employment of hTL inhibitors as chemotherapeutics is limited by their heavy side effects (Guterres and Villanueva, 2020), which could be reduced by a dual-target based approach. At the moment, compounds of this subclass were designed by combining the azidothymidine moiety, which binds to hTL (Strahl and Blackburn, 1994), with different hCAI scaffolds (i.e., benzesulfonamide, coumarine and solfocumarine) through a linker containing the 1,2,3-triazole ring (Berrino et al., 2020) (Figure 1E). Inhibition experiments indicated that they strongly inhibited hCA XII (K_I_ values in the range 2.8–78.9 nM), whereas some of them possessed medium−high inhibition potency against hCA IX (K_I_ = 3.7–8047.1 nM). The most effective inhibitors of hCA IX and XII were also tested for their antitelomerase properties in PC3 and HT-29 cells, revealing that they were able to highly reduce hTL activity (Berrino et al., 2020; Plyasova et al., 2021). Further experiments on compound **8b**, the top-performing hCA IX inhibitor of the series, and compound **8c**, possessing the most favourable hCA XII/hCA IX inhibition ratio, highlighted that these molecules suppressed hTL activity in human colorectal cancer cell lines, while a prolonged incubation resulted in telomere shortening, cell cycle arrest, replicative senescence, and apoptosis. Finally, *in vivo* Colo-205 mouse xenograft studies demonstrated antitumor activity only for compound **8c** thus confirming the high theraupetic potential of this type of molecules (Plyasova et al., 2021). Structural analysis on three key representatives of hCA-hTL dual inhibitors in complex with hCA II showed that sulfonamide moiety was coordinated to Zn(II), while an intricate network of polar and hydrophobic interactions contributed to stabilize inhibitor binding and modulate its orientation within hCA active site (Berrino et al., 2020; Plyasova et al., 2021). In particular, it was suggested that the linker moiety could play a key role in determining hCA isoform selectivity affecting the inhibitor tail orientation (Figure 2D).

## 5 Dual targeting compounds for treating neurological diseases

Very recently two interesting studies employing dual targeting hCAIs for the treatment of diverse neurological diseases, i.e., oxaliplatin-induced neuropathy (OINP) and Glucose Transporter Type 1 Deficiency Syndrome (GLUT1-DS), were reported by Angeli et al. (2023a, 2023b).

For the management of OINP (Lehky et al., 2004), the Authors proposed a series of molecules that were capable of modulating both hCAs and the Transient Receptor Potential Vanilloid 1 (TRPV1) (Angeli et al., 2023b). TRPV1 recently assumed importance as a potential analgesic target (Wang, 2008; Benítez-Angeles et al., 2020) and its activation represents a promising strategy for pain management (Bamps et al., 2021; Iftinca et al., 2021). These compounds were designed introducing the sulfonamide moiety into the TRPV1 antagonist modulator SB-705498 (Figure 1F) and operating various modifications, such as substitutions of aromatic rings, bio-isosteric switch between ureido and thioureido linkers and the introduction of stereocenters (compounds **9**, **10a-10b**, **11a-11b** in Figure 1F). The resulting molecules were tested *in vitro* against physiologically relevant hCA isoforms (I, II, IV, VII, IX, XII) and TRPV1, showing to be effective toward hCAs, whereas selected items reported moderate agonism of TRPV1. Moreover, the data obtained showed that the presence of (*R*)- or (*S*)-stereocenters within the synthesized compounds did not appear to significantly impact the activity of both targets. The X-ray structures of the adducts of two enantiomers (*R*)-**10a** and (*S*)-**10b** with hCA II were also reported (Figure 2E). Although the compounds showed comparable effectiveness towards hCA II, with K_I_ values of 6.7 and 4.9 nM, respectively, their crystallographic structures revealed some differences in their binding mode. Indeed, both molecules preserved the typical benzenesulfonamide interactions with the catalytic Zn(II) and active site residues, but revealed significant differences in the conformation of the two tails, occupying distinct hydrophobic subpockets that are separated by the Phe131 aminoacid (Figure 2E). Interestingly, *in vivo* studies of the top-performing compounds ((*R*)-**9**, (*R*)-**10a**, (*R*)-**11a**, and (*S*)-**11b** in Figure 1F) revealed prolonged pain-relief effects in a mouse model of OINP (Angeli et al., 2023b). The Authors conclude that the dual activity of these compounds as mild TRPV1 agonists and potent hCAIs represents a promising strategy for managing OINP symptoms, like pain.

In the case of GLUT1-DS, Angeli et al. (2023a) developed a series of compounds with dual targeting capabilities, designed to target either hCA isoforms or GLUT1 transporters as key targets of GLUT1-DS associated seizures (Klepper et al., 2020).

GLUT1-DS is a mutational based genetic disorder resulting in the aberrant expression of the transporter GLUT1, thus affecting its ability to intake glucose (Klepper et al., 2020) and leading to cognitive impairment, and drug resistant seizures. This series of compounds was characterized by a hCA inhibiting moiety (i.e., sulfonamide) and a GLUT1 substrate, such as D-glucose and D-galactose (Winum et al., 2009; Ung et al., 2016) (Figure 1G). All compounds were evaluated *in vitro* on human-expressed CAs, revealing a heterogeneous inhibition pattern. Crystallographic studies were performed to determine the 3D structure of hCA II in complex with compound **12a**, having a particularly favourable binding affinity for this isoform (K_I_ value of 7.5 nM) (Figure 2F). The primary sulfonamide moiety of compound **12a** maintained the classical anchoring to Zn(II), whereas the glucosyl hydroxyl groups formed hydrogen bonds with Asn62, Asn67, and Gln92, thus justifying the high binding affinity.

Interestingly, almost all the compounds tested demonstrated to be effective activators of GLUT1 by *in vitro* glucose uptake assays. The Authors suggested that these compounds act as stabilizers of GLUT1 functional clusters on cellular membranes. Selected compounds (**12b**, **12c**, **13** in Figure 1G) were further investigated for their ability to abolish the occurrence of seizures *in vivo* by means of the induced maximal electroshock seizure model, revealing that derivative **12c** was particularly effective in suppressing seizures at a specific dosage range, without inducing any side effects. This finding supports a novel pharmacological approach for managing diseases associated with GLUT1-DS.

## 6 Conclusion

hCAs are well-recognized human targets for the treatment of serious diseases including glaucoma, inflammatory conditions, cancer, and neurological disorders. In order to identify effective hCAIs, many compounds exploring a wide range of chemotypes, such as sulfonamides and their bioisosters, coumarines and carboxylic acids, have been so far reported.

However, a promising strategy for new drug development is based on the identification of dual-target inhibitors able to affect different pathways. In fact, many ongoing efforts in the field of medicinal chemistry are directed towards the innovative emerging paradigm of dual-targeting approach since it overcomes multiple limits of the classic ‘one-molecule one-target’ strategy, including drug resistance, dose toxicity and unpredictable pharmacokinetic properties. In this context, molecules capable of interacting simultaneously with hCAs and another protein target have been recently designed. Identification of drug–target interactions plays an important role in drug discovery and development, shedding light on the molecular determinants responsible for the binding of an inhibitor with its targets. Since there is little structural data currently available on the complexes that hCAs form with molecules possessing a dual-target mechanism of action, further studies are needed.
